# The DSM: mindful science or mindless power? A critical review

**DOI:** 10.3389/fpsyg.2014.00602

**Published:** 2014-06-17

**Authors:** Bassam Khoury, Ellen J. Langer, Francesco Pagnini

**Affiliations:** ^1^Department of Psychology, Harvard University, CambridgeMA, USA; ^2^Department of Psychology, Catholic University of MilanMilan, Italy; ^3^Niguarda Ca′ Granda HospitalMilan, Italy

**Keywords:** DSM, mindful, mindless, mindfulness, normality, pathology, utility

## Abstract

In this paper we review the Diagnostic and Statistical Manual of mental health (DSM), its scientific bases and utility. The concepts of “normality,” “pathology,” and boundaries between them are critically reviewed. We further use the concepts of mindfulness and mindlessness, and evidence from cognitive and social sciences to investigate the DSM clinical and social impact and we argue against its assigned overpower. We recommend including alternative perspectives to the DSM, such as mindfulness and positive psychology. We also argue for including mindfulness training in psychiatric residency and clinical psychology programs.

## THE DSM

The Diagnostic and Statistical Manual of Mental Disorders (DSM) is considered the most important document for the diagnosis and the classification of mental disorders. Despite the existence of alternative diagnostic criteria and approaches [e.g., International Statistical Classification of Diseases (ICD), Psychodynamic Diagnostic Manual (PDM)], the DSM criteria remain the gold standard for mental health diagnosis. It is published by the American Psychiatric Association (APA), whose initial objective was to establish a nosology of mental disorders that can constitute a common language among clinicians, researchers, health insurance companies, and the pharmaceutical industry. Five versions and two revisions of the manual were published since 1952, the last one being its fifth edition published in May 18, 2013. Since its first draft, the DSM went through many modifications. For example the number of proclaimed mental disorders went from 108 in its first version (1952), to 182 in DSM-II published in 1968, to 265 in DSM-III published in 1980, followed by its revision DSM-III-R in 1987 with 292 diagnoses, to 354 categories in the DSM-IV published in 1994, followed by its revision DSM-IV-R in 2000 with no significant modifications, and lastly the DSM-5, which did not change significantly the number of disorders but rather the criteria (or thresholds) of diagnoses, leading to a potential inflation of some diagnosis up to 28% (Keely et al., 2008; Corcoran and Walsh, 2010; Millon et al., 2010; Frances, 2013; Greenberg, 2013). Along with the increasing number of disorders, the manual went from 130 pages in its first edition to 886 pages in its fourth and 991 in its current edition, this was accompanied with a substantial growth in the price, sales, and revenues of DSM for the APA reaching between $5 and $6 million annually, almost 10% of its global revenue (Greenberg, 2013, p. 110). The income from the DSM for the APA is very trivial in comparison with the income for the pharmaceutical industry, which exceeds $18 billion a year in sales of psychotropic medication, with more than $12 billion a year from antidepressants sales only (Frances, 2013, p. 89). Beyond the numbers and facts concerning the DSM, which were extensively addressed elsewhere (e.g., Frances, 2013; Greenberg, 2013), there is an ongoing debate inside the scientific and clinical communities about the DSM science and utility. This papers aims to shed more light on the science and utility of the DSM categories, while suggesting other possibilities and alternative approaches.

## THE SCIENCE AND UTILITY OF THE DSM

A central aim of the DSM taskforce was to set appropriate cut-off points between what is considered “normal” from what is “pathological,” in order to treat the individuals belonging to the latter category. The concept of pathology is an aprioristic definition, originally based on statistical distribution (the “S” in DSM), with an arbitrary decision that is not scientifically driven (Frances, 2013, p. 24). In fact, the criteria set for cut-offs between “normal” and “pathological” (e.g., number of symptoms, frequency and duration of symptoms, and nature/duration of dysfunction associated with the proclaimed disorder) are also “arbitrary” and subjective because there is no laboratory test or biological markers to set the boundary between “normal” and “pathological.” In addition, there is not scientific link between basic science (e.g., cognitive, neurological and social science) and clinical psychiatry. Second, cultures differ dramatically in their conception of normality; what is “normal” in one culture can be considered “abnormal” or “pathological” in another one. In addition, there are significant within-and-between cultural differences in the manner in which diagnostic categories are interpreted and diagnostic labels are used (e.g., Zubin, 1967; Rosenhan, 1973; Crow, 1986; Zinbarg et al., 1994; Keller et al., 1995). For example, the APA defined homosexuality as a mental disorder until the DSM-II in 1973 (Greener, 2013). Third, manifestations (named symptoms in the DSM) and behaviors are contextually embedded and vary dramatically according to the situation, for example, aggressive behavior can be well-adapted in one context but not in another one. Even if the DSM partially addresses this issue, subtle contextual differences may create several diagnostic biases. Last, when normality is set in relationship to the ability of understanding reality (i.e., in the definition of psychosis and dissociative disorders), it is arguable that reality itself is constantly changing and hard or even impossible to grasp.

From another side, studies regarding the prevalence of mental disorders using the DSM categories did not provide coherent results, as it would be expected. In fact, there are several differences when comparing the prevalence rates obtained. For example, retrospective studies suggested that the annual prevalence of DSM psychiatric disorders is around 30% in adults younger than 55 years and projected a lifetime prevalence rate of 50% for a psychiatric disorder by 75 years of age (Kessler et al., 2005). However, these rates seem to be an underestimation as evidence of forgetting is common for recall beyond 6 months (e.g., Harlow and Linet, 1989; Pillemer and White, 1989; Angold and Costello, 1996). Therefore, prospective studies can be more accurate (Moffitt et al., 2010; Copeland et al., 2011). Using prospective methodology, the cumulative prevalence of DSM-IV defined categories among 1037 individuals during a 15-year prospective longitudinal study (between age 18 and 32) yielded to 50% for an anxiety disorder, 41% for depression, 32% for alcohol dependence, and 18% for cannabis dependence (Moffitt et al., 2010). Another prospective longitudinal study assessing 1420 participants for nine times from 9 through 21 years of age yielded to 61.1% for a well-specified psychiatric disorder. An additional, 21.4% had met the criteria for a not otherwise specified disorder only, increasing the cumulative prevalence for any disorder to 82.5% (Copeland et al., 2011). In the youngest cohort, the cumulative prevalence for any disorder was higher than 90% (Copeland et al., 2011). In addition, there is evidence of a mounting epidemic of mental disorders in the last 15 years. In fact, childhood bipolar disorder increased 40-fold (Moreno et al., 2007), autism increased by 20-fold, attention deficit hyperactivity disorder (ADHD) has tripled (Bloom et al., 2011), and adult bipolar disorder doubled (Ketter, 2010). These data add more confusion about the efficacy of the DSM in delineating “normality” from “pathology” as some of these statistics suggest that almost all of the population has mental disorders.

The DSM-5 taskforce aimed to address this problem by implementing a dimensional aspect to the DSM that was supposed to reflect a continuum view of mental disorders rather than a categorical one. However, not only the taskforce failed to fully implement dimensions in the new DSM but also kept its categorical aspect and reduced the thresholds for many diagnostic criteria, which can lead to a wide increase in pathologizing previously considered “normal” individuals (Frances, 2013) making the population almost totally saturated with mental disorders. These arguments taken together raise serious questions regarding the science behind the DSM, specifically its reliability, validity and clinical utility.

In fact multiple reviews questioned the reliability and the validity of many DSM categories. For example, Blom and Oberink (2012) found that the construct validity of DSM-IV post-traumatic stress disorders (PTSD) in children and adolescents varies among different criteria: where some are highly valid (e.g., stressor criterion), while others are not (e.g., avoidance, detachment from others, and difficulty falling or staying asleep). In addition, some non-DSM criteria (e.g., guilt) had better validity than existing ones (e.g., avoidance and emotional numbing criterion). Vieta and Phillips (2007) argued that the content, concurrent, discriminant, and predictive validity of bipolar disorder are problematic suggesting a need to improve and refine diagnostic criteria. Woo and Rey (2005) found that the validity of the inattentive and hyperactive-impulsive subtypes of ADHD is not fully supported in the DSM-IV pointing to a deficit in data on treatment of the inattentive and hyperactive-impulsive subtypes. In conjunction with these results, a meta-analysis involving 546 studies concluded that DSM-IV ADHD subtypes do not identify discrete subgroups with sufficient long-term stability to justify the classification of distinct forms of the disorder. In summary, many reviews were highly critical of the DSM, while few others supported some DSM criteria [e.g., validity of atypical depression Lam and Stewart (1996); cross-cultural construct validity of ADHD in children and adolescents Willcutt (2012)].

These results taken together are particularly disappointing especially that the DSM went through multiple modifications and ameliorations in the last sixty years. In that line, Laungani (2002) argued that the popularity and extensive use of the DSM is not an indication of its reliability or validity. A theory, according to Lakatos (1970) may be true, even if no one believes in it, and it may be false, even if everyone believes in it. In addition, a low congruence was found between DSM-IV and International Diagnostic Interview (ICD-10) for many psychiatric categories including schizophrenia, schizoaffective disorder, bipolar disorder and depression (e.g., Cheniaux et al., 2009). Moreover, the rising number of individuals qualifying for at least one psychiatric disorder during lifetime renders the boundaries between “normal” and “pathological” illusive and nullifies the DSM validity and its principal reason for existence.

A second aim for the DSM is supposed to be clinical, i.e., setting a common language among clinicians in order to encourage collaboration and improving treatments for individuals with a diagnosis of a mental disorder. However, it is questionable why the DSM labels are needed to further clinical help for patients. Among the arguments for the use of psychiatric labels is that they are simple, easy, clear, quick, and convenient to use. If this argument is true, it is equally problematic as a simple and quick label can be automatically used without in-depth mental processing. This is particularly precarious specifically with the previously shown prevalence of psychiatric diagnoses among the general population.

Many scholars and clinicians have argued that psychiatric labels serve only the interests of clinicians and their professional associations (e.g., APA) as well as the pharmaceutical industry (Greenberg, 2013), whereas these labels can have devastating effects of the individuals receiving them (e.g., Frances, 2013, p. 109). In fact, labels can create self-fulfilling prophecies (Rosenthal and Fode, 1963), reducing expectations, ambitions, and changing other’s perceptions and behaviors toward the individual carrying the label (Smith, 2002). Ben-Zeev et al. (2010) identified three types of stigma resulting from DSM diagnoses: public stigma, self-stigma, and label avoidance (Corrigan and Watson, 2002; Corrigan et al., 2004). Public stigma is the phenomenon of large social groups endorsing negative stereotypes about, and subsequently acting against, a stigmatized group: in this case, people with a diagnosis of mental disorder. Self-stigma is the loss of self-esteem and self-efficacy that occurs when the individuals internalize public stigma, which may prevent them from pursuing their life goals (Corrigan, 2006). Label avoidance is the phenomenon leading individuals to avoid mental health services in order to avoid the deleterious impact of a stigmatizing label. In addition, three processes can further exacerbate the stigma associated with psychiatric labels (Ben-Zeev et al., 2010). The first is groupness defined as the degree to which a collection of people is perceived as a unified or meaningful entity (Campbell, 1958; Hamilton and Sherman, 1996). Diagnosis distinguishes people with a mental disorder from the general population and adds to the salience of their groupness (Link and Phelan, 2001). Research has also shown a non-specific prejudice against people who have a psychiatric disorder compared with people with other health conditions (Weiner et al., 1988; Corrigan et al., 2000). In addition, diagnostic labels can serve as priming for automatic negative stereotypes (e.g., Devine, 1989; Bargh et al., 1996). Negative attitudes were also shown to be automatically activated among therapists (Abreu, 1999). Moreover, diagnostic labels of severe mental illness such as schizophrenia and psychosis seem to worsen the level of prejudice and this is even worse following a first psychotic episode (Crisp et al., 2000; Phelan et al., 2000; Birchwood et al., 2007; Lolich and Leiderman, 2008; Reed, 2008). The second is homogeneity, where out-groups members are seen more homogeneous than in-groups (Tajfel, 1978; Rothbart et al., 1997; Ashton and Esses, 1999). Categorization or groupness was also shown to increase negative stereotypes against out-group members (Link and Phelan, 2001); however, there can be causal bidirectional relationship between both (Yzerbyt et al., 1997; Crawford et al., 2002). The third is stability, meaning the traits that describe group members are believed to remain relatively stable and unchanging (Anderson, 1991; Kashima, 2000). Stability also supports the idea that psychiatric diagnoses are unchanging and that individuals are less likely to overcome them in comparison with those with physical illnesses (Weiner et al., 1988; Corrigan et al., 2000). This pessimistic view of stability is even worse in the case of severe mental illness (e.g., psychosis and schizophrenia; Harding and Zahniser, 1994). Taken together, these processes can lead to an overgeneralization error, where all members of a group are expected to manifest the same characteristics attributed to that group (Ben-Zeev et al., 2010). In addition psychiatric diagnoses when delivered rigidly, and unconditionally (without being related to specific contexts) are likely to yield to internal, stable, incontrollable and global negative attributions about the self, modifying self-concept and leading to a sense of hopelessness and learned helplessness (Seligman, 1975), which ironically was shown to be related to another popular DSM category, that is, major depressive disorder (MDD; e.g., Maiden, 1987; Healy and Williams, 1988; Duman, 2010; Vollmayr and Gass, 2013).

Taking into consideration the negative effects of psychiatric labels, which seem to outweigh any claimed benefits, it is legitimate to reconsider their clinical utility and their advantages compared to direct descriptions of the phenomenological experience of individuals seeking psychiatric or psychological help. For example, simple and direct experiential descriptors namely, emotions of sadness, worry, fear, anger, disgust, terror, and lack of energy, motivation, pleasure, and hope as well as specific thought patterns (e.g., rumination, over-generalization, and pessimism), physical sensations (e.g., fatigue, exhaustion, palpitations, fainting, and sleeplessness), cognitive processing (e.g., inattention, distraction, and memory loss), and behaviors (e.g., avoidance, isolation, or aggression) are common among individuals and provide better insight for appropriate treatment than abstract psychiatric constructs (e.g., depression, anxiety, borderline, and psychosis). In addition, the attention of the clinician must be particularly directed toward the distress and suffering experienced by the individual and toward the mental/behavioral processes that maintain and exacerbate the suffering (e.g., mind-wandering, identification with one’s own thoughts, acting in opposite ways of personal values, and lack of self-acceptance and compassion).

In conjunction with their clinical utility, DSM categories are been argued to be particularly useful for pharmacological treatment. Perhaps this is the best use of psychiatric diagnoses. However, scientific research remains unclear and controversial about the benefits of a specific type of medication for a specific psychiatric diagnosis and psychotropic medications such as antidepressants and antipsychotics are been prescribed for a multitude of psychiatric disorders, including sleeping, anxiety, depression, irritability, eccentricity, temper tantrums in youth, and crankiness of old age (Frances, 2013, p. 105). In addition, the psychotropic prescribing industry is being one of the fast growing, financing a large part of DSM related research activities and financing APA itself, leading to important questions regarding the clinical necessity of such growth and its dubious ethics specifically that some of these drugs can be dangerous causing massive obesity, diabetes, heart disease, and a shortened life span (Laungani, 2002; Frances, 2013, p. 89; Greenberg, 2013). Beside these concerns, and despite of the little scientific knowledge regarding the mechanisms of actions of most of the prescribed medications, empirical findings support their utility for many individuals in specific contexts. According to that, it will be irresponsible and unethical to advise individuals stopping their medications; however, more scientific and ethical boundaries must be implemented in order to reduce unnecessary prescription and to fully explain to individuals the state of knowledge regarding the possible benefits as well as negative short and long-term effects of these medications.

Another proclaimed utility for the DSM categories is the advancement of clinical research. This is true in the scope that a large number of studies would use DSM categories. However, most of the research outcomes are measured using quantitative data, i.e., raw numbers resulting from administering specific clinical measures, e.g., Beck Depression Inventory (Beck et al., 1961, 1996; Beck and Streer, 1987), Beck Anxiety Inventory (Beck and Streer, 1993), and qualitative data, mostly obtained through clinical interviews.

A last utility for the DSM is proclaimed to be legal as many of the diagnoses have implications within the legal system (e.g., paraphilia). However, despite their use today, it is not advisable for health science to be part of the legal debates in courts as it further undermines its primary role of treating individuals (Dawes, 1994). Other utilities are within the financial, political and social domains (e.g., health insurance); however, a review of these benefits is outside the scope of the current paper.

In summary, the DSM, when thoroughly investigated, yields to some support regarding its reliability but leads to serious questions about its validity, utility, and ethics. These findings cannot justify the overuse of DSM in mental health neither the power nor authority assigned to the DSM categories besides being only of financial and sociopolitical reasons.

## THE DSM: MINDFUL OR MINDLESS?

The discussion about the DSM could be seen in the light of the concepts of mindfulness/mindlessness (Langer, 1989, 1997, 2012). Mindlessness is described as a default style of cognitive functioning in which individuals process cues from the environment in a relatively automatic but inflexible manner, without reference to novel aspects of these cues (Langer and Piper, 1987). By default, old categories and previously made distinctions are relied on uncritically, leading to rigid behavior that is rule governed rather than rule guided. In contrast, mindfulness is described as a general style or mode of functioning through which individuals actively engage in reconstructing the environment by creating new categories or distinctions, and seeking multiple perspectives, thus directing attention to new contextual cues that may be consciously controlled or manipulated as appropriate (Langer, 1989, 1997, 2012). There is growing evidence for the adaptive influence of mindful functioning on learning, health, and social behavior and, conversely, for the deleterious effects of mindlessness (e.g., Langer et al., 1978, 1985, 2012; Langer and Newman, 1979; Langer and Piper, 1987; Langer, 1989, 1992a,b, 1997, 2000, 2009, 2012).

It is been suggested that a mindful approach to language, where individuals are made aware of alternative perspectives and conceptions of what is being said or written, leads to more control and better outcomes (Langer and Piper, 1987; Langer, 1992a). The DSM is an example from the opposite side, being written using an absolute unconditional language leading to a narrow perspective of the complex human mental conditions and states by labeling some of these conditions as mental disorders. In fact, the DSM embraced a single intrapersonal definition of mental conditions, considering them as internal flaws, due to biological defects, maladaptation to the society, and/or personality difficulties, minimizing or denying at times the implication of external (environmental, interpersonal and social) factors and trivializing the lack of support facing a large number of “normal” individuals in Western societies today. For example, many of the cognitive and functional limitations perceived in the elderly can be due to non-adapted environment, an environment designed by young adults for young adults and not for the elderly (Langer, 2009). In the social sciences, this phenomenon is known as the fundamental attribution error, defined as the tendency to place a heavy emphasis on internal characteristics to explain someone else’s behavior in a given situation, rather than considering external situational factors (Jones and Harris, 1967; Ross, 1977a). Fundamental attribution error is further committed when attention is fully directed toward the behavior of someone else, specifically when it is evaluated as deviant (Smith and Miller, 1979; Robinson and McArthur, 1982; Lassiter et al., 2002), when the evaluator is in an automatic (mindless) mental mode (Winter and Uleman, 1984; Uleman, 1987; Moskowitz, 1993; Newman, 1993; Carlston and Skowronski, 1994), or the evaluator lacks energy or motivation (e.g., Gilbert, 1989), and is more present in individualistic cultures (e.g., North-American) in comparison with collectivist cultures (e.g., Asian; Miller, 1984; Michael and Kaiping, 1994; Morris and Peng, 1994; Masuda and Nisbett, 2001; Langdridge and Trevor, 2004). In addition, the DSM mindlessly focuses on the inabilities or limitations of the diagnosed individuals without outweighing their shortcomings with their abilities, talents and resilience. Moreover, the DSM does not acknowledge change across time, but rather focuses on stable traits (e.g., in defining personality disorders). Being blind to the reality of continuous change is another aspect of mindlessness (Bodner and Langer, 2001). The single perspective of the DSM can exacerbate prejudice toward already negatively stereotyped individuals (e.g., individuals with a diagnosis of mental retardation; Reiss, 2000) and can contribute in justifying the under-investment of governments and health agencies in underprivileged individuals (Laungani, 2002), by rending their suffering as their own fault and responsibility and by labeling them as “abnormal and deviant” (Reiss, 2000; Pilgrim and Rogers, 2005). Such mindless application of diagnostic criteria is consistent with data on illusory correlations and psychodiagnostic tests (e.g., Chapman and Chapman, 1967, 1969; Dowling and Graham, 1976; Mirels, 1976), where illusory correlations (i.e., non-existing, over-evaluated or even opposite correlations) seem to persist even with the passage of time blinding the diagnostician in the face of contradictory reality. This phenomenon was present not only in projective and non-empirical psychological tests (e.g., Wheeler-Rorschach; Chapman and Chapman, 1969) but equally in empirical test batteries (e.g., Minnesota Multiphasic Personality Inventory – MMPI; Dowling and Graham, 1976) and was shown to be more pronounced among more experienced diagnosticians than novice ones (Dowling and Graham, 1976). A possible explanation of this phenomenon is premature cognitive commitment (Chanowitz and Langer, 1981), where previously created categories are available for mindless use (e.g., Langer and Imber, 1979), even if information is presented in a single instance (e.g., Chanowitz and Langer, 1980). This phenomenon was shown to worsen with time (i.e., with the exposure to previously learned material) as in the case of experienced diagnosticians (Dowling and Graham, 1976). This is particularly true when information is presented in an absolute, unconditional, authoritarian, and stable manner (Langer and Piper, 1987), which is the case of most psychodiagnostic tests and the DSM diagnostic criteria. Absolute diagnostic categories encourage habit, reduce uncertainty and unpleasant insecurity among diagnosticians but at the same time render it difficult for them to produce alternative, novice perceptions, distinctions or categories, making them mindlessly following previously established rules and categories without doubting or questioning these rules. In addition, medical residents and graduate psychology students are less prone to learn when taught with unconditional material (e.g., using DSM categories; Langer, 1997, 2000). Moreover, when presented with absolute diagnostic labels from a trusted figure of scientific authority (e.g., psychiatrist, psychologist or other mental health professionals), the individual receiving the label, even though, she is more mindful about her condition, will most likely give-up personal control accepting the label mindlessly and resigning powerlessly to its consequences, which can be devastative in many cases. In such dynamic of clinician power, authority and knowledge versus unpowered and diagnostic-naive “patient”, it is highly likely that the latter will experience a lack of personal control, self-determination, in addition to the shame, stigma and infringement to self-dignity and self-esteem, with powerful physical and mental negative consequences. A counter-mechanism is to increase the control of individuals on their own health whether physical or mental, which was shown to have powerful positive consequences (Langer and Rodin, 1976; Rodin and Langer, 1977).

According to social science, the DSM can be considered as a perfect example of actor–observer bias (Kelley, 1967; Nisbett et al., 1973; Ross, 1977b; Watson, 1982; Jones and Nisbett, 1987; Gilbert, 1995), which refers to the tendency of emphasizing internal, dispositional causes (e.g., personality traits) when explaining others’ behavior but considering own behavior to stem primarily from external, situational factors (e.g., being under stress). Malle et al. (2007) and Malle (2004, 2006) suggested a contribution of cognitive access and beliefs (e.g., the lack of context-specific information and the use of heuristics) among observers due to motivational differences with actors. This definition when applied to a non-contextually based DSM, and considering the possible motivation of the DSM users, suggests an increase in mindless use of DSM categories among mental health professionals. Laungani (2002) argued that conceptions of normality derived through the use of the DSM are, to a large extent, based on notions of social conformity. When clinicians are referring only to social conventions and previously learned information (e.g., DSM categories) in making their mind about their perceived “mentally ill” patients, they are less prone to seek novel information and less likely to have an open and curious orientation toward their patients’ environment. Such mindless orientation towards the past may lead to misdiagnosis, mistreatment, and seriously compromise the therapeutic alliance between the professional and the individual seeking help.

In summary, the DSM is not only a mindless categorization of mental conditions, but also the way in which it is taught to mental health professionals encourages a mindless use of the DSM categories. That is similar to a widely diffused approach toward medical data, which is collected, justified, accepted, and used uncritically and mindlessly (Langer, 2012).

## BEYOND THE DSM

Few attempts were made in Western sciences to understand human mental conditions and suffering beyond diagnostic categories. For example, Millon et al. (2010, Chap. 30) tried to extend the criteria of borderline personality disorder (as defined in the DSM) by including evolutionary, social, and cultural factors recognizing the diversity of the syndrome. However, the authors did not consider the abilities and resilience of individuals with the proclaimed syndrome.

An alternative model was also proposed in positive psychology (e.g., Peterson and Seligman, 2004) categorizing a set of 24 personality traits of “character strength and virtues.” Among these traits are: creativity, curiosity, open-mindedness, wisdom, love, kindness, social intelligence, forgiveness, humility, self-regulation, and gratitude. It is noteworthy that many of these traits are related to mindfulness, whether defined according to Eastern tradition (e.g., Baer et al., 2004, 2006; Hutcherson et al., 2008) or to Western science (e.g., Langer, 1989, 1997, 2005, 2009; Langer and Moldoveanu, 2000a; Haigh et al., 2011). A mindful constructivist approach to the mental health sciences must be developed by recognizing the cultural and social embeddedness of the individual rather than using any single definition or set of criteria for human conditions (Langer and Moldoveanu, 2000b). This approach may be the key for the development of mindful clinical psychology and psychiatry. The data suggest that mindfulness is related to better outcomes for patients of therapists practicing Zen meditation (Grepmair et al., 2007). Once having realized that mindfulness can be a key element in providing better health services, the next question will be how to encourage health care professionals to learn and practice mindfulness.

Reiss (2000) suggested that mindfulness has motivational bases; the most important appears to be a desire to learn (curiosity). By engaging in mindful thinking, people can satisfy their desire for curiosity. Other motivational components are: a low need for an order (allowing for a greater creativity and mental flexibility), and a need for independence, defined as a desire for self-reliance, allowing the individual to rely on his/her own ideas and to think more freely and independently from conventions or external pressures (e.g., peer pressure and authority). These motivational bases must be used in designing and developing training programs for psychiatric residents and psychology graduate students encouraging them to uphold an open, curious and multiple perspective (mindful) attitude while investigating the conditions of their patients. Mindfulness can also be learned by imitating the behavior of a mindful therapist or supervisor. This further supports teaching mindfulness to supervisors and health decision makers.

## CONCLUSION

The DSM shows both scientific and clinical limits, its wide use and the blind approval of its categories and criteria must be carefully reconsidered. When the DSM is used, it must be accompanied with alternative perspectives, emphasizing different aspects of human suffering including social, environmental, and political dimensions. Clinicians should also be mindful about the strengths and abilities of their patients and should emphasize their resilience rather than their perceived deficits. Other disciplines, namely cognitive and social sciences should be used as a solid foundation of a new scientifically driven clinical psychology and psychiatry. It is time that psychological science moves from the mindless investigation of mental disorders and psychopathology to the mindful science of mental states and consciousness.

## Conflict of Interest Statement

The authors declare that the research was conducted in the absence of any commercial or financial relationships that could be construed as a potential conflict of interest.
